# Serum Adropin Levels and Body Mass Composition in Kidney Transplant Recipients—Are There Sex Differences?

**DOI:** 10.3390/diagnostics13172768

**Published:** 2023-08-26

**Authors:** Josipa Radić, Sanja Lovrić Kojundžić, Andrea Gelemanović, Marijana Vučković, Danijela Budimir Mršić, Daniela Šupe Domić, Maja Dodig Novaković, Mislav Radić

**Affiliations:** 1 Department of Nephrology and Dialysis, University Hospital of Split, 21000 Split, Croatia; josiparadic1973@gmail.com (J.R.); mavuckovic@kbsplit.hr (M.V.); 2 Department of Internal Medicine, School of Medicine, University of Split, 21000 Split, Croatia; mislavradic@gmail.com; 3 Department of Diagnostic and Interventional Radiology, University Hospital of Split, 21000 Split, Croatia; danijelabudimir@gmail.com; 4 School of Medicine, University of Split, 21000 Split, Croatia; 5 Department of Health Studies, University of Split, 21000 Split, Croatia; daniela.supe.domic@ozs.unist.hr; 6 Biology of Robusteness Group, Mediterranean Institute for Life Sciences (MedILS), 21000 Split, Croatia; andrea.gelemanovic@gmail.com; 7 Department of Medical Laboratory Diagnostics, University Hospital of Split, 21000 Split, Croatia; 8 Department of Radiology, General Hospital Šibenik, 22000 Šibenik, Croatia; dodig.maja@gmail.com; 9 Department of Rheumatology and Clınıcal Immunollogy, University Hospital of Split, 21000 Split, Croatia

**Keywords:** adropin, kidney transplant recipients, nutritional status, body composition

## Abstract

Adropin is a secretory peptide that regulates glucose, lipid, and protein metabolism, which is closely related to obesity, insulin resistance, dyslipidemia, and atherogenesis. The serum adropin level is related to sex and depends upon nutritional preferences. This study aims to determine the association between serum adropin levels and body composition parameters in kidney transplant recipients (KTRs), especially emphasizing sex differences. Our case–control study involved 59 KTRs (28 postmenopausal women and 31 men) who were divided into two groups according to sex, and each group of those KTRs was further divided into higher or lower adropin values than the mean value in each sex group. Univariate regression showed a negative association of adropin levels with most anthropometric and body composition parameters in men’s KTRs. Contrary to this, the serum adropin level was negatively associated only with phase angle in postmenopausal female KTRs. Multivariate regression showed that skeletal muscle mass and phase angle were the only negative predictors in women’s KTRs, whereas in men, negative predictors were BMI and body water. These findings imply that adropin could have a different impact on metabolic homeostasis in KTRs regarding sex and could be considered a negative predictor of body composition in KTRs.

## 1. Introduction

Adropin is a recently identified regulatory protein, encoded by energy balance-related genes (Enho) and involved in the maintenance of energy homeostasis [1,2]. This peptide hormone is mainly expressed in the liver and brain but is also present in other tissues such as the heart, cerebellum, lung, kidney, muscles, and pancreas [3,4,5].

Recent studies have shown that adropin is involved in the regulation of glucose, lipid, and protein metabolism, which is closely related to obesity, insulin resistance, dyslipidemia, and atherogenesis [2,3,6]. Animal studies confirmed that adropin levels in serum are low in mice with high-fat diet-induced obesity and that adropin knock-out mice (AdrKO) displayed increased adiposity despite normal food intake [7].

However, studies investigating plasma adropin concentrations in humans have indicated some differences between sexes. For example, women have lower serum adropin levels compared to men [2]. Although most investigations have shown a negative correlation between body mass index (BMI) and adropin levels, this rule has sometimes not been shown to be statistically significant or applicable to both sexes. In the study of St-Onge, high plasma adropin concentrations were found only in male patients and associated with the lean phenotype at a younger age [8]. On the contrary, this association is completely reversed to an increased risk of obesity later in life [8].

Moreover, the relationship between plasma adropin concentrations and the levels of low-density lipoprotein cholesterol (LDL-C) is also sex-dependent and is only found in men [9]. This effect on cholesterol homeostasis is more evident in overweight to obese men patients and limited to atherogenic LDL-C without influence on very-low-density lipoprotein cholesterol (VLDL-C) or high-density lipoprotein cholesterol (HDL-C) levels [9]. Furthermore, the association of adropin levels with nutritional preferences was only confirmed in female patients. Plasma adropin levels showed a positive correlation with fat intake [8] and a negative correlation with the consumption of carbohydrates [10].

Several studies have investigated adropin, focusing on the female population in the generative period. A study based on polycystic ovary syndrome (PCOS), which is associated with obesity, dyslipidemia, and insulin resistance, showed lower serum and follicular fluid (FF) adropin levels in PCOS women compared to control patients of similar age and BMI [11]. Contrary to this, a study based on a younger androgenic PCOS group did not find any correlation between adropin or any other anthropometric parameters, but they observed a positive correlation between adropin and androstenedione levels [12].

A recent study investigating the role of adropin in autoimmune disease—primary Sjogren syndrome (pSS), which is more common in women—showed that these patients have significantly higher serum adropin levels compared to healthy controls [13]. Also, adropin was positively correlated with HDL-C and anti-SSA/Ro52 antibodies in patients with pSS.

Accordingly, there is no study investigating the role of adropin and parameters of body composition in immunocompromised populations as in KTRs. Therefore, our study aimed to assess serum adropin levels and body mass composition parameters in KTRs and to assess sex differences between men and postmenopausal women who are not influenced by hormonal disturbances.

## 2. Materials and Methods

### 2.1. Study Design and Population

This cross-sectional study was conducted at the Outpatient Clinic for Clinical Nutrition, Nephrology and Dialysis Division, Internal Medicine Clinic, University Hospital of Split, Croatia, between July 2020 and October 2020.

The study comprised 59 KTRs, aged over 18, with functional graft, no mobility issues, and follow-up for more than a year following kidney transplantation (28 women and 31 men). The exclusion criteria were active inflammatory or malignant disease, history of stroke and myocardial infarction, implanted pacemaker or cardioverter defibrillator, stents, or limb amputation.

### 2.2. Medical History and Clinical and Laboratory Parameters

Baseline clinical data included the patient’s age and sex, smoking status, presence of chronic kidney disease, cardiovascular risk factors (hyperlipidemia, arterial hypertension, and diabetes mellitus), presence of cardiovascular and cerebrovascular disease, duration, and type of dialysis treatment before kidney transplantation. 

In terms of laboratory parameters, all study participants received standard peripheral blood sampling on the same day as the body composition and blood pressure measurements. Peripheral venous blood samples were collected following overnight fasting and handled according to standard laboratory practice by an experienced medical biochemist blinded to group assignments. The conventional hematological and biochemical parameters were analyzed on the same day. The samples for adropin analysis were centrifuged and maintained at −80 °C until analysis.

We measured the following parameters: urea (mmol/L), creatinine (mmol/L), uric acid (mmol/L), serum albumin (g/L), phosphates (mmol/L), C-reactive protein (CRP; mg/L), calcium (mmol/L), glucose (mmol/L), triglycerides (TG; mmol/L), total cholesterol (TC; mmol/L), LDL-C, (mmol/L), erythrocytes (10^12^), hemoglobin (g/L), mean cellular volume (MCV; fL), sodium (mmol/L), potassium (mmol/L), and estimated glomerular filtration rate (eGFR; mL/min/1.73 m^2^) using Chronic Kidney Disease Epidemiology Collaboration (CKD-EPI). A complete blood count was obtained using a hematology analyzer (Advia 120, Siemens, Erlangen, Germany).

Serum adropin levels were determined using a commercially available dual enzyme-linked immunosorbent assay (ELISA) kit (Phoenix Pharmaceuticals, Burlingame, CA, USA) according to the manufacturer’s instructions. The test range was 0.3–20 ng/mL and sensitivity of 0.08 ng/m, and inter-assay and intra-assay coefficients of variation (CV) within the probe were less than 10%.

### 2.3. Body Composition and Anthropometric Measurements

Each participant’s body composition was analyzed using bioelectrical impedance measurement (BIA) with a Tanita MC-780 Multi Frequency Segmental Body Analyzer (Tokyo, Japan). BIA included analysis of these data: body mass (kg), muscle mass (kg and %), skeletal muscle mass (kg and %), fat mass (kg and %), fat-free mass (kg and %), visceral fat, trunk fat mass (kg and %), skeletal muscle mass (kg and %), sarcopenic muscle index (SMI), phase angle (PhA (◦)), total body water (TBW, kg), extracellular water (EW, kg) and intracellular water (IW, kg). 

All patients were requested to refrain from eating or drinking anything for at least three hours before the measurement, to urinate right before the analysis, and to abstain from alcohol, excessive eating or drinking, and excessive training for at least one day before the body composition assessment [14]. Anthropometric measurements included information on each study participant’s height, weight, BMI, waist circumference, mid-upper arm circumference, and waist to height ratio (WHtR).

### 2.4. Central Blood Pressure and Arterial Stiffness Measurement

Using an Agedio B900 (IEM, Stolberg, Germany) oscillometry-based equipment, peripheral and central blood pressure and arterial stiffness were measured. The correct-sized cuff was chosen and precisely placed based on the upper arm circumference. All participants were analyzed while calmly seated, with their backs and arms supported, feet flat on the floor, their legs uncrossed, and their bladders empty. We collected information on peripheral systolic blood pressure (pSBP, mmHg), peripheral diastolic blood pressure (pDBP, mmHg), peripheral mean arterial pressure (pMAP, mmHg), peripheral pulse pressure (pPP), central systolic blood pressure (cSBP, mmHg), central diastolic blood pressure (cDBP, mmHg), central mean arterial pressure (cMAP, mmHg), central pulse pressure (cPP), mmHg), and pulse wave velocity (PWV; m/s).

### 2.5. Statistical Analysis

The normality of the data was first evaluated using the Shapiro–Wilks test. In cases where the data were normally distributed, it was presented with mean and standard deviation (SD), whereas if the data were not normally distributed, it was presented with median and interquartile range (IQR). Categorical data were presented as numbers with percentages. To test the differences between the groups, the chi-square test, T-test, or Mann–Whitney test were applied as appropriate. To evaluate the predictors of serum adropin levels, first, a univariate linear regression was performed, after which all parameters with *p*-values less than 0.1 were entered into the LASSO regression model, which was used to select the most relevant variables. Finally, a multivariate linear regression model was applied with selected variables after LASSO regression to identify the strongest predictors for serum adropin levels and to obtain beta coefficients, standard errors, and *p*-values.

## 3. Results

### 3.1. Baseline Clinical, Anthropometric, Laboratory, Body Composition, and Blood Pressure Parameters of the Study Population

The study included 59 KTRs of which 31 (53%) were men and 28 (47%) were women. All studied KTR women were in the postmenopausal period. 

Table 1 presents data about baseline clinical characteristics; anthropometric, laboratory, body composition, and blood pressure parameters; and sex differences in all those parameters. Generally, most of the sex differences were observed between body composition parameters. Men had significantly more muscle mass and body water, and women had significantly more fat content. Also, several sex differences were shown regarding laboratory parameters (hemoglobin, cholesterol, and phosphates). Men’s and women’s KTRs did not differ in other observed parameters, including serum adropin levels, as shown in Table 1.

To determine whether there are sex differences depending on higher and lower adropin values, we divided both sex groups into two subgroups depending on the mean adropin value (≤mean value and >mean value). Therefore, we obtained two subgroups in female KTRs regarding the adropin value: those with lower adropin values (n= 11 (39%)) and women with higher adropin values (n = 17 (61%)). We used the same methodology to categorize men into two subgroups: those with lower adropin values (N = 14 (45%)) and male patients with higher adropin values than the mean value (n = 17 (55%)).

There was no statistically significant difference within the adropin subgroups in the time since transplantation, the type of dialysis, comorbidities, and smoking status. We observed significant differences in both sexes between the adropin subgroups for body weight (in women *p* = 0.015; men *p* = 0.012), BMI (women *p* = 0.035; men *p* = 0.005), and waist circumference (in women *p* = 0.020; men *p* = 0.036). Therefore, we observed significant differences between the sexes in the relation of adropin levels to body composition. We found only in male KTRs that serum adropin level was significantly related to fat tissue mass (*p* = 0.003), the percentage of fat tissue (*p* = 0.009), and visceral fat (*p* = 0.021). Contrary to this, the serum adropin levels in female KTRs were associated with overall muscle mass and skeletal muscle mass (*p* = 0.044 and *p* = 0.033, respectively), as shown in Table 2. Considering the sex differences in the relation of adropin to the proportion of water, we found a significant difference in the mass of water in men (*p* = 0.019), while in women, this difference was noticed only in the percentage of body water (*p* = 0.039). As shown in Table 2, regarding the relation of adropin levels to laboratory parameters, we found significant differences for creatinine (*p* = 0.035), triglycerides (*p* = 0.037), and urate (*p* = 0.028) in the male group. Among female KTRs, a significant difference was detected only for potassium (*p* = 0.026).

### 3.2. The Association of Serum Adropin Levels and Clinical, Anthropometric, Laboratory, Body Composition, and Blood Pressure Parameters

As presented in Table 3, univariate linear regression analysis showed that age (*p* = 0.022) and PhA (*p* = 0.025) were significant predictors of serum adropin levels in female KTRs.

In contrast, in male KTRs, serum adropin levels were negatively associated with weight (*p* < 0.001), BMI (*p* < 0.001), upper arm circumference (*p* = 0.010), and waist circumference (*p* = 0.010). Considering the body composition parameters, we found a significant negative association for most of the parameters for male KTRs. Male KTRs showed a significant negative association of adropin with the percentage of fat mass (*p* = 0.005), fat-free mass (*p* = 0.001), visceral fat (*p* = 0.021), muscle mass (*p* = 0.001), skeletal muscle mass, (*p* = 0.002), body water (*p* = 0.019), the percentage of body water (*p* = 0.015), PhA (*p* = 0.002), and trunk visceral fat (*p* = 0.002). When observing blood pressure parameters, the only significant and negative association was found for pDBP (*p* = 0.019) and cDBP (*p* = 0.041) in male KTRs, as shown in Table 3.

### 3.3. Multivariate Linear Regression Analysis Showed Association of Adropin with Age and BMI

**Figure 1:**
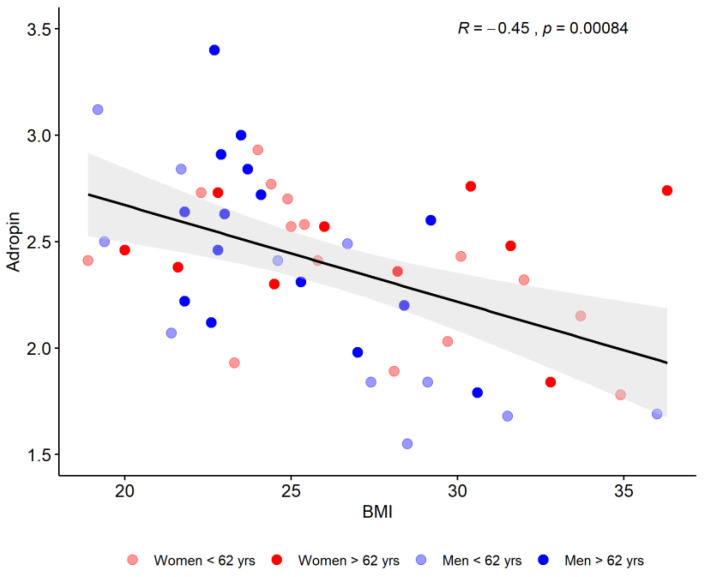
Associations between serum adropin levels and BMI regarding age and sex. Abbreviations: BMI—body mass index.

The whole sample showed only a significant positive correlation of adropin values with age (*p* = 0.020) and a negative value with BMI (*p* < 0.001) as shown in Figure 1.

After selecting the strongest predictors for serum adropin levels following the multivariate LASSO regression model, for female KTRs, the only significant negative predictors were skeletal muscle mass (kg; beta = −0.024, SE = 0.011, *p* = 0.043) and PhA (beta = −0.207, SE = 0.099, *p* = 0.046), whereas for male KTRs, the only significant negative predictors were BMI (beta = −0.403, SE = 0.072, *p* < 0.001) and body water (kg; beta = −0.022, SE = 0.008, *p* = 0.003).

## 4. Discussion

The main result of our study in KTRs showed that most of the anthropometric and body composition parameters, as well as some of the laboratory and blood pressure parameters, differed between the high and low serum adropin groups, which was more pronounced in men compared to postmenopausal women. In men, most of these anthropometric and body composition parameters also remained negative predictors of serum adropin levels. In male KTRs, adropin was not a predictor of almost all measured parameters, except for PhA. Multivariate analysis showed that independent predictors were also different between men and women. These results confirmed substantial sex differences in adropin levels in the KTRs cohort.

The study confirmed that lower adropin levels were related to higher body weight and fat content, which was estimated with the following anthropometric parameters: weight, waist circumference, WHtR, and BMI. Although the mentioned parameters differed between high and low adropin groups in both sexes, they were significant predictors of adropin levels only in men. The negative association between adropin, weight gain, and BMI was well confirmed in the previous studies conducted in different cohorts and settings [15,16]. The adropin levels were shown to be lower in overweight and obese vs. normal weight populations, suggesting a possible role of this hormone in the development of obesity. BIA has become established over the past four decades as a widespread technique for the assessment of body composition. In our study, adropin was associated with several body composition parameters estimated by BIA, such as fat mass, visceral fat, body, and extracellular water in men, while in women, only skeletal muscle and muscle mass were significantly higher in the group with lower adropin levels. Similar results were obtained in a study by Yosaee et al. [15], which compared patients with metabolic syndrome, obese, and normal weight patients, including more than 86% of men of younger age in the population sample. It seems that higher body fat amounts are related to a negative impact on serum adropin levels. However, the exact role of adropin in the pathogenesis of obesity is not fully understood. It remains unclear whether overexpression of adropin merely delays body weight gain or prevents it. Also, the exact mechanism of the lowering of serum adropin concentrations while raising excess body fat amounts remains unexplained. Laboratory parameters examined in our study were generally not associated with serum adropin levels, especially in women. Previous studies have shown that high adropin levels can improve lipid metabolism, and a negative association between adropin and cholesterol levels, LDL - C and TG, and a positive association with HDL- C have been described [17,18]. Low adropin was associated with dyslipidemia in hemodialysis patients only in unadjusted analysis [19], but after excluding confounders, the lipid levels were shown not to be related to adropin levels. Similarly, regression analysis in a study by Hu et al. in patients with diabetes mellitus type 2 showed no association between lipids and adropin levels [20]. In our cohort, no association was found either, except for the case of the independent negative prediction of TG levels of adropin in men. This suggests that an association may exist, although it may be weak. Future studies will better explain the involvement of adropin in lipid homeostasis.

Blood pressure parameters generally showed the least association with serum adropin levels, except for cDBP and pDBP in male KTRs, which differed between high and low adropin level groups and remained negative predictors of adropin levels. Previous studies have shown that adropin concentrations are lower in hypertension [21]. Adropin was propped to be an inductor of nitric oxide (NO) production, which acts as a vasodilator [22]. It was also shown that adropin is involved in controlling the functions of endothelial cells [5]. Adropin may influence blood pressure by protecting endothelial function, and low adropin levels are related to endothelial dysfunction [23], which could partially explain the connection.

In female KTRs, skeletal muscle mass and PhA were found to be negative predictors of adropin. The PhA is a body composition measure of cellular stability. It was shown to be lower in obesity and metabolic syndrome. In a previous study, PhA was positively associated with body fat percent, free fat mass, upper arm circumference, and muscle mass [24], thus explaining the connection. Previous research showed in general a weaker association between female sex and adropin levels compared to male sex, which could be partially explained by the influence of estrogens [25]. However, in our cohort of KTRs postmenopausal women, in whom hormones could not influence adropin levels, the weaker association of adropin with all parameters remained in women.

Finally, kidney transplantation is the treatment of choice for end-stage renal disease (ESRD). However, KTRs are at risk of developing metabolic disorders. The increasing use of immunosuppressive therapy, which is potentially diabetogenic and atherogenic, can worsen pre-existing medical conditions or induce the development of other problems including bone disease, infections, and malignancies [26]. The pathogenesis of cardiovascular disease as a major cause of death in this population is multifactorial and can be reduced through numerous lifestyle modifications. The modification and adjustment of drug therapy by reducing the use of glucocorticoids, calcineurin inhibitors, or other drugs in patients with major risk factors for cardiovascular disease, as well as the consistent and timely treatment of diabetes, hypertension, and/or hyperlipidemia and anemia can also reduce cardiovascular morbidity in the KTRs population [27].

So far, there are several studies conducted on ESRD patients, which showed lower levels of adropin in patients receiving dialysis treatment compared to controls [18,19]. The study based on a rat model of chronic kidney failure showed that adropin had a protective effect on inflammation and chronic kidney damage progression [28]. In another study, reduced levels of adropin were associated with renal dysfunction in patients with type 2 diabetes mellitus, and adropin was proposed to be used as a biomarker for the early diagnosis of diabetic nephropathy [29]. The latest study confirmed that type 2 diabetics with chronic heart failure with lower adropin values were more likely to develop chronic kidney disease [30]. Moreover, recent studies have proposed a possible immunomodulatory effect of adropin. Also, autoimmune disorders such as pSS, or rheumatoid arthritis were shown to be associated with lower adropin levels [13,31]. This shows how low adropin level accompanies these conditions and has a potential role in the pathogenesis of the disease. To the best of our knowledge, there are no studies on immunocompromised populations that were investigated like in our study. Immunosuppressants administered to KTRs could potentially interfere with and disrupt the protective immunomodulatory effects of adropin, but the exact mechanism remains to be elucidated.

The main strength of the study, to the best of our knowledge, is that this was the first research that investigated associations of serum adropin levels and body composition in an immunocompromised KTRs cohort. Furthermore, it is important to note that this KTRs cohort is specific for its low incidence of diabetes, obesity, and cardiovascular disease. It is important to underline that women in our cohort were all postmenopausal, which is another confirmation of the sex dependence of adropin in the KTRs population. The results of this research will provide new insights into the possible role of serum adropin in the pathophysiology of cardiovascular risk in these susceptible immunocompromised KTRs. The limitations of this study are a relatively small number of KTRs and a lack of healthy control. The cross-sectional study design did not allow for the definition of any causal relations.

## 5. Conclusions

The present study shows that higher adropin levels are associated with different metabolic abnormalities in male KTRs compared to postmenopausal women. This confirms sex-dependent adropin differences in the immunocompromised KTRs population.

## Figures and Tables

**Table 1:** Baseline and observed parameter characteristics of the study population including sex differences.

Predictor	Women	Men	*p*
	n = 28 (47%)	n = 31 (53%)	
Transplantation (years), median (IQR)	5 (7.5)	5 (7)	0.993
PD, n (%)	13 (48.15)	9 (31.03)	0.394
HD, n (%)	12 (44.44)	18 (62.07)	0.394
PD + HD, n (%)	2 (7.41)	2 (6.9)	0.394
Dialysis (years), median (IQR)	1.75 (4)	3 (3.58)	0.379
Age (years), median (IQR)	60 (13.5)	65 (11.5)	0.168
Arterial hypertension—no n (%)	4 (14.29)	5 (16.67)	1.000
Arterial hypertension—yes n (%)	24 (85.71)	25 (83.33)	1.000
Diabetes Mellitus—no, n (%)	23 (85.19)	24 (77.42)	0.676
Diabetes Mellitus—yes, n (%)	4 (14.81)	7 (22.58)	0.676
Cardiovascular Disease—no, n (%)	22 (84.62)	24 (80)	0.920
Cardiovascular Disease—yes, n (%)	4 (15.38)	6 (20)	0.920
Cerebrovascular Disease—no, n (%)	25 (96.15)	27 (90)	0.710
Cerebrovascular Disease—yes, n (%)	1 (3.85)	3 (10)	0.710
Nonsmoker, n (%)	10 (40)	12 (50)	0.652
Former smoker, n (%)	7 (28)	7 (29.17)	0.652
Smoker, n (%)	8 (32)	5 (20.83)	0.652
Anthropometric parameters			
Height (cm), mean (SD)	165.04 (5.97)	178.38 (8.67)	<0.001
Weight (kg), mean (SD)	73.9 (14.15)	80.34 (15.17)	0.123
BMI (kg/m^2^), mean (SD)	27.07 (4.73)	25.19 (4.02)	0.132
Waist circumference, mean (SD)	97.78 (14.75)	100.22 (11.49)	0.567
WHtR, mean (SD)	0.59 (0.09)	0.56 (0.06)	0.191
Laboratory parameters			
Albumin (g/L), mean (SD)	41.5 (3.05)	40.75 (3.43)	0.421
Calcium (mmol/L), median (IQR)	2.47 (0.22)	2.43 (0.16)	0.416
CRP (mg/L), median (IQR)	2.5 (4.22)	2.25 (3.65)	0.769
Erythrocyte count (×10^12^), mean (SD)	4.48 (0.47)	4.64 (0.61)	0.077
Fasting blood glucose (mmol/L), median (IQR)	5.1 (0.75)	5.2 (0.85)	0.566
Hemoglobin (g/L), median (IQR)	130.44 (10.8)	139.1 (12.93)	0.009
Potassium (mmol/L), mean (SD)	4.02 (0.46)	4.21 (0.56)	0.169
Cholesterol (mmol/L), mean (SD)	6.13 (1.06)	5.35 (1.27)	0.028
Creatinine (mmol/L), median (IQR)	112 (57)	123 (40)	0.231
LDL-C (mmol/L), mean (SD)	3.66 (0.89)	3.18 (1.05)	0.107
MCV (fL), mean (SD)	88.79 (5.38)	88.71 (5.54)	0.954
Sodium (mmol/L), median (IQR)	141.85 (1.69)	141.5 (1.56)	0.470
Phosphate (mmol/L), mean (SD)	1.1 (0.22)	0.97 (0.23)	0.042
Triglycerides (mmol/L), median (IQR)	1.6 (1.1)	1.9 (1.67)	0.652
Uric acid (mmol/L), mean (SD)	387.44 (67.36)	389.86 (70.72)	0.896
Urea (mmol/L), median (IQR)	9.15 (3.65)	9.6 (2.6)	0.679
eGFR (mL/min/1.73 m^2^), mean (SD)	48.73 (21.28)	53.92 (19.38)	0.344
Adropin, mean (SD)	2.37 (0.36)	2.37 (0.47)	0.974
Body composition parameters			
Fat mass (kg), median (IQR)	21.8 (13.7)	12.6 (11.55)	0.003
Fat mass (%), mean (SD)	29.31 (7.95)	17.1 (7.52)	<0.001
Fat free mass (kg), mean (SD)	51.51 (7.82)	66.52 (9.22)	<0.001
Visceral fat, mean (SD)	7.4 (2.9)	9.62 (3.63)	0.021
Metabolic age (years), median (IQR)	50 (13)	50.5 (12)	0.817
Muscle mass (kg), mean (SD)	48.9 (7.44)	63.12 (8.94)	<0.001
Skeletal muscle mass (kg), median (IQR)	26 (4.4)	37.8 (7.63)	<0.001
Skeletal muscle mass (%), mean (SD)	37.43 (5.55)	46.52 (7.5)	<0.001
Body mass (kg), mean (SD)	2.61 (0.38)	3.31 (0.43)	<0.001
Body water (kg), mean (SD)	36.52 (5.62)	46.85 (6.84)	<0.001
Body water (%), mean (SD)	50.1 (5.7)	58.41 (6.14)	<0.001
Phase angle (°), mean (SD)	5.26 (0.6)	5.35 (0.91)	0.682
ECW, mean (SD)	16.21 (2.37)	19.45 (2.24)	<0.001
ICW, median (IQR)	19.4 (3.3)	28.2 (5.72)	<0.001
Trunk visceral fat, mean (SD)	9.76 (4.65)	8.22 (5.72)	0.307
Blood pressure parameters			
pSBP (mmHg), mean (SD)	132.67 (18.38)	137.74 (14.05)	0.254
pDBP (mmHg), mean (SD)	86.61 (11.77)	87.48 (12.78)	0.793
pMAP (mmHg), mean (SD)	108.32 (12.92)	110.57 (11.76)	0.537
pPP (mmHg), mean (SD)	48.32 (14.67)	50.8 (11.97)	0.533
cSBP (mmHg), mean (SD)	126.38 (16.25)	130.36 (13.42)	0.376
cDBP (mmHg), mean (SD)	87.14 (12.27)	89.02 (12.01)	0.603
cMAP (mmHg), mean (SD)	100.22 (12.29)	102.8 (11.26)	0.465
cPP (mmHg), mean (SD)	35.8 (11.26)	39.24 (11.01)	0.303
AIx, mean (SD)	21.72 (13.77)	19.98 (12.22)	0.654
PWV(m/s), mean (SD)	8.83 (1.61)	9.15 (1.77)	0.502

Abbreviations: n—number, PD—peritoneal dialysis, HD—hemodialysis, BMI—body mass index, WHtR—waist to height ratio, CRP—C-reactive protein, LDL—C —low density lipoprotein cholesterol, MCV—mean cellular volume, eGFR—estimated glomerular filtration rate using CKD-EPI, ECW—extracellular water, ICW—intracellular water, p—peripheral, c—central, SBP—systolic blood pressure, DBP—diastolic blood pressure, MAP—mean arterial pressure, PP—pulse pressure, AIx—augmentation index, PWV—pulse wave velocity.

**Table 2:** Difference regarding adropin categories in each sex group.

	Total Number of KTRs with Measured Adropin (n = 59)					
	Women (n = 28)			Men (n = 31)		
	Adropin ≤ Mean Value (n = 11)	Adropin > Mean Value (n = 17)	*p*	Adropin ≤ Mean Value (n = 14)	Adropin > Mean Value (n = 17)	*p*
Time since transplantation (years) median (IQR)	3.5 (3.75)	6 (8)	0.236	5 (8)	5 (6.5)	0.567
Dialysis type, n (%)						
PD	5 (50)	8 (47.06)	0.893	6 (46.15)	3 (18.75)	0.257
HD	4 (40)	8 (47.06)	0.893	6 (46.15)	12 (75)	0.257
PD+HD	1 (10)	1 (5.88)	0.893	1 (7.69)	1 (6.25)	0.257
Dialysis duration (years) median (IQR)	1.25 (1.5)	2 (4)	0.517	2 (3.5)	4 (3)	0.256
Age (years) median (IQR)	54.45 (9.98)	60.71 (9.53)	0.108	62.5 (16.75)	65 (13)	0.361
Presence of chronic kidney disease, n (%)						
No	4 (40)	3 (17.65)	0.409	3 (23.08)	6 (37.5)	0.666
Yes	6 (60)	14 (82.35)	0.409	10 (76.92)	10 (62.5)	0.666
Presence of arterial hypertension, n (%)						
No	2 (18.18)	2 (11.76)	1.000	2 (15.38)	3 (17.65)	1.000
Yes	9 (81.82)	15 (88.24)	1.000	11 (84.62)	14 (82.35)	1.000
Presence of diabetes mellitus, n (%)						
No	9 (90)	14 (82.35)	1.000	10 (71.43)	14 (82.35)	0.770
Yes	1 (10)	3 (17.65)	1.000	4 (28.57)	3 (17.65)	0.770
Presence of cardiovascular disease, n (%)						
No	8 (88.89)	14 (82.35)	1.000	11 (78.57)	13 (81.25)	1.000
Yes	1 (11.11)	3 (17.65)	1.000	3 (21.43)	3 (18.75)	1.000
Presence of cerebrovascular disease, N (%)						
No	8 (88.89)	17 (100)	0.742	12 (85.71)	15 (93.75)	0.903
Yes	1 (11.11)	NA	0.742	2 (14.29)	1 (6.25)	0.903
Smoking status, N (%)						
Nonsmoker	2 (22.22)	8 (50)	0.290	5 (41.67)	7 (58.33)	0.713
Former smoker	4 (44.44)	3 (18.75)	0.290	4 (33.33)	3 (25)	0.713
Smoker	3 (33.33)	5 (31.25)	0.290	3 (25)	2 (16.67)	0.713
Anthropometric parameters						
Height (cm), mean (SD)	167.11 (6.75)	163.88 (5.35)	0.199	178.75 (6.52)	178.07 (10.41)	0.847
Weight (kg), mean (SD)	82.82 (12.2)	68.89 (12.9)	0.015	88.15 (17)	73.65 (9.67)	0.012
BMI (kg/m^2^), mean (SD)	29.69 (4.04)	25.59 (4.54)	0.035	27.47 (4.27)	23.24 (2.58)	0.005
Upper arm circumference (cm), median (IQR)	30.67 (3.57)	28.57 (3.5)	0.179	28 (10)	27 (2.75)	0.510
Waist circumference (cm), mean (SD)	106.44 (8.78)	92.21 (15.35)	0.020	105.78 (12.01)	94.67 (8.17)	0.036
WHtR, mean (SD)	0.64 (0.05)	0.56 (0.09)	0.043	0.59 (0.05)	0.54 (0.06)	0.074
Laboratory parameters						
Albumin (g/L), mean (SD)	41.78 (2.77)	41.33 (3.29)	0.738	40.18 (3.03)	41.17 (3.74)	0.478
Calcium (mmol/L), median (IQR)	2.53 (0.16)	2.41 (0.15)	0.055	2.44 (0.17)	2.42 (0.16)	0.645
CRP (mg/L), median (IQR)	3.85 (5.75)	2.1 (3.72)	0.218	3.9 (5.3)	1.9 (2.55)	0.612
Erythrocyte count (×10^12^), mean (SD)	4.6 (0.45)	4.39 (0.4)	0.229	4.53 (0.42)	4.9 (0.65)	0.091
Fasting blood glucose (mmol/L), median (IQR)	5.1 (0.78)	5.11 (0.66)	0.967	5 (1)	5.3 (0.95)	0.258
Hemoglobin (g/L), median (IQR)	132.9 (11.05)	129 (10.71)	0.375	134.38 (12.71)	142.94 (12.16)	0.076
Potassium (mmol/L), mean (SD)	4.27 (0.4)	3.87 (0.44)	0.026	4.6 (0.9)	3.95 (0.45)	0.061
Cholesterol (mmol/L), mean (SD)	5.92 (1.97)	6.2 (0.67)	0.619	5.6 (1.53)	5.14 (1)	0.365
Creatinine (mmol/L), median (IQR)	105.5 (84)	113 (40)	0.581	149 (59)	116 (34.5)	0.035
LDL-C (mmol/L), mean (SD)	3.3 (0.8)	3.7 (1)	0.363	3.34 (1.29)	3.05 (0.83)	0.500
MCV (fL), mean (SD)	87.29 (4.82)	89.73 (5.65)	0.269	89.3 (3.37)	88.19 (6.99)	0.607
Sodium (mmol/L), median (IQR)	141.12 (1.55)	142.33 (1.67)	0.121	141.64 (1.86)	141.4 (1.35)	0.710
Phosphate (mmol/L), mean (SD)	1.06 (0.27)	1.12 (0.18)	0.478	1.06 (0.27)	0.82 (0.2)	0.016
Triglycerides (mmol/L), median (IQR)	1.8 (1.1)	1.6 (0.9)	0.591	2.65 (1.55)	1.16 (1.33)	0.037
Uric acid (mmol/L), mean (SD)	385.2 (78.47)	388.76 (62.48)	0.897	421.23 (63.17)	364.38 (67.8)	0.028
Urea (mmol/L), median (IQR)	9 (4.7)	9.3 (3.2)	1.000	11.42 (4.08)	9.32 (2.2)	0.087
eGFR (mL/min/1.73 m^2^), mean (SD)	54.54 (25.77)	45.32 (18.14)	0.286	46.68 (20.87)	59.8 (16.44)	0.069
Adropin, mean (SD)	2.01 (0.26)	2.6 (0.16)	0.000	1.94 (0.24)	2.73 (0.27)	0.000
Body composition parameters						
Fat mass (kg), median (IQR)	26.69 (8.76)	19.99 (8.72)	0.079	20.1 (8.82)	9.45 (6.72)	0.003
Fat mass (%), mean (SD)	31.8 (7.42)	27.91 (8.12)	0.249	21.65 (6.15)	13.85 (6.82)	0.009
Fat free mass (kg), mean (SD)	56.3 (4.2)	47.95 (5.67)	0.044	70.75 (6.95)	63.49 (9.67)	0.055
Visceral fat, mean (SD)	8.44 (3)	6.81 (2.76)	0.182	11.6 (2.27)	8.21 (3.83)	0.021
Metabolic age (years), median (IQR)	50.56 (14.29)	48.25 (12.76)	0.682	51.5 (7.5)	49.5 (14.25)	0.907
Muscle mass (kg), mean (SD)	53.5 (4)	45.5 (5.4)	0.044	67.25 (6.61)	60.18 (9.42)	0.054
Skeletal muscle mass (kg), mean (SD)	29.1 (3.1)	25.2 (2.75)	0.033	38.97 (4.85)	35.09 (6.95)	0.144
Skeletal muscle mass (%), median (IQR)	36.19 (5.39)	38.13 (5.68)	0.412	43.03 (3.31)	49.01 (8.71)	0.052
Body mass (kg), mean (SD)	2.8 (0.2)	2.45 (0.28)	0.056	3.5 (0.34)	3.17 (0.44)	0.060
Body water (kg), mean (SD)	40.2 (2.8)	33.9 (3.82)	0.039	49.76 (5.47)	44.77 (7.14)	0.077
Body water (%), mean (SD)	48.42 (5.31)	51.05 (5.86)	0.278	55.03 (4.08)	60.82 (6.33)	0.019
Phase angle (°), median (IQR)	5.49 (0.72)	5.14 (0.5)	0.164	5.72 (0.79)	5.09 (0.92)	0.095
ECW, mean (SD)	17.67 (2.33)	15.39 (2.03)	0.018	20.68 (1.93)	18.58 (2.08)	0.020
ICW, mean (SD)	21.7 (2.3)	18.8 (2.07)	0.033	29.08 (3.62)	26.19 (5.18)	0.144
ECW/ICW, mean (SD)	0.8 (0.07)	0.8 (0.07)	0.911	0.72 (0.04)	0.72 (0.09)	0.796
Trunk visceral fat, median (IQR)	12.08 (4.16)	8.46 (4.51)	0.060	12.45 (6.03)	4.75 (4.77)	0.003
Blood pressure parameters						
pSBP (mmHg), mean (SD)	132.68 (18.47)	132.66 (18.92)	0.997	139.19 (11.87)	136.29 (16.26)	0.594
pDBP (mmHg), mean (SD)	88.41 (11.09)	85.38 (12.42)	0.521	91.14 (14.48)	83.82 (10.02)	0.132
pMAP (mmHg), mean (SD)	108.28 (13.09)	108.34 (13.25)	0.991	111.82 (10.24)	109.32 (13.49)	0.630
pPP (mmHg), mean (SD)	43 (15.39)	51.31 (13.84)	0.179	45.64 (12.54)	55.95 (9.23)	0.040
cSBP (mmHg), mean (SD)	122.22 (17.47)	128.72 (15.61)	0.348	130.25 (9.65)	130.45 (16.62)	0.973
cDBP (mmHg), mean (SD)	89.72 (12.44)	85.69 (12.33)	0.442	93.4 (12.76)	85.05 (10.25)	0.113
cMAP (mmHg), mean (SD)	100.57 (12.85)	100.03 (12.39)	0.918	105.68 (10.56)	100.18 (11.72)	0.275
cPP (mmHg), mean (SD)	30.83 (13.19)	38.59 (9.32)	0.099	36 (12.09)	42.18 (9.53)	0.207
PWV (m/s), median (IQR)	8.1 (1.71)	9.24 (1.45)	0.091	9.4 (2.15)	9.6 (1.4)	0.460

Abbreviations: KTRs—kidney transplant recipients, n—number, PD—peritoneal dialysis, HD—hemodialysis, BMI—body mass index, WHtR—waist to height ratio, CRP—C-reactive protein, LDL—C —low density lipoprotein cholesterol, MCV—mean cellular volume, eGFR—estimated glomerular filtration rate using CKD-EPI, ECW—extracellular water, ICW—intracellular water, p—peripheral, c—central, SBP—systolic blood pressure, DBP—diastolic blood pressure, MAP—mean arterial pressure, PP—pulse pressure, PWV—pulse wave velocity.

**Table 3:** Comparison between men and women related to significant predictors that determine the serum adropin level.

Predictor	Univariate Linear Regression					
	Women (n = 28)			Men (n = 31)		
	Beta	SE	*p*	Beta	SE	*p*
Age (years)	0.015	0.006	0.022	0.011	0.006	0.105
Anthropometric parameters						
Weight (kg)	−0.006	0.005	0.169	−0.023	0.005	0.000
BMI (kg/m^2^)	−0.023	0.013	0.098	−0.082	0.018	0.000
Upper arm circumference (cm)	−0.010	0.019	0.608	−0.066	0.023	0.010
Waist circumference (cm)	−0.005	0.005	0.306	−0.026	0.009	0.010
Laboratory parameters						
Phosphate (mmol/L)	0.236	0.329	0.480	−0.814	0.375	0.039
Triglycerides (mmol/L)	−0.019	0.084	0.822	−0.177	0.074	0.026
Body composition parameters						
Fat mass (kg)	−0.007	0.007	0.348	−0.039	0.009	0.000
Fat mass (%)	−0.003	0.008	0.742	−0.037	0.012	0.005
Fat free mass (kg)	−0.012	0.008	0.165	−0.034	0.009	0.001
Visceral fat	−0.003	0.023	0.897	−0.065	0.026	0.021
Muscle mass (kg)	−0.012	0.009	0.164	−0.036	0.009	0.001
Skeletal muscle mass (kg)	−0.021	0.014	0.147	−0.043	0.014	0.006
Body mass (kg)	−0.234	0.168	0.176	−0.739	0.198	0.001
Body water (kg)	−0.017	0.011	0.150	−0.044	0.013	0.002
Body water (%)	0.003	0.012	0.808	0.040	0.015	0.015
Phase angle	−0.238	0.100	0.025	−0.300	0.100	0.006
ECW	−0.036	0.027	0.199	−0.151	0.035	0.000
ICW	−0.028	0.018	0.146	−0.058	0.019	0.006
Trunk visceral fat	−0.010	0.014	0.499	−0.061	0.014	0.000
Blood pressure parameters						
pDBP (mmHg)	−0.004	0.006	0.494	−0.017	0.007	0.019
cDBP (mmHg)	−0.005	0.005	0.334	−0.018	0.008	0.041

Abbreviations: n—number, BMI—body mass index, ECW—extracellular water, ICW—intracellular water, p—peripheral, c—central, DBP—diastolic blood pressure.

## Data Availability

Data are available upon request to the corresponding author via e-mail.
